# Associations between active travel and weight, blood pressure and diabetes in six middle income countries: a cross-sectional study in older adults

**DOI:** 10.1186/s12966-015-0223-3

**Published:** 2015-05-20

**Authors:** Anthony A. Laverty, Raffaele Palladino, John Tayu Lee, Christopher Millett

**Affiliations:** Department of Primary Care & Public Health, Imperial College London, London, UK; Department of Public Health, University Federico II of Naples, Naples, Italy; Room 322, Reynolds Building, St Dunstan’s Road, W6 8RP London, UK

## Abstract

**Background:**

There is little published data on the potential health benefits of active travel in low and middle-income countries. This is despite increasing levels of adiposity being linked to increases in physical inactivity and non-communicable diseases. This study will examine: (1) socio-demographic correlates of using active travel (walking or cycling for transport) among older adults in six populous middle-income countries (2) whether use of active travel is associated with adiposity, systolic blood pressure and self-reported diabetes in these countries.

**Methods:**

Data are from the WHO Study on Global Ageing and Adult Health (SAGE) of China, India, Mexico, Ghana, Russia and South Africa with a total sample size of 40,477. Correlates of active travel (≥150 min/week) were examined using logistic regression. Logistic and linear regression analyses were used to examine health related outcomes according to three groups of active travel use per week.

**Results:**

46.4 % of the sample undertook ≥150 min of active travel per week (range South Africa: 21.9 % Ghana: 57.8 %). In pooled analyses those in wealthier households were less likely to meet this level of active travel (Adjusted Risk Ratio (ARR) 0.77, 95 % Confidence Intervals 0.67; 0.88 wealthiest fifth vs. poorest). Older people and women were also less likely to use active travel for ≥150 min per week (ARR 0.71, 0.62; 0.80 those aged 70+ years vs. 18–29 years old, ARR 0.82, 0.74; 0.91 women vs. men).

In pooled fully adjusted analyses, high use of active travel was associated with lower risk of overweight (ARR 0.71, 0.59; 0.86), high waist-to-hip ratio (ARR 0.71, 0.61; 0.84) and lower BMI (−0.54 kg/m^2^, −0.98;− 0.11). Moderate (31–209 min/week) and high use (≥210 min/week) of active travel was associated with lower waist circumference (−1.52 cm (−2.40; −0.65) and −2.16 cm (3.07; −1.26)), and lower systolic blood pressure (−1.63 mm/Hg (−3.19; −0.06) and −2.33 mm/Hg (−3.98; −0.69)).

**Conclusions:**

In middle-income countries use of active travel for ≥150 min per week is more common in lower socio-economic groups and appears to confer similar health benefits to those identified in high-income settings. Efforts to increase active travel levels should be integral to strategies to maintain healthy weight and reduce disease burden in these settings.

**Electronic supplementary material:**

The online version of this article (doi:10.1186/s12966-015-0223-3) contains supplementary material, which is available to authorized users.

## Introduction

Recent evidence has highlighted a secular trend of increasing Body Mass Index (BMI) across countries at all stages of development [1]. Obesity and overweight are now serious concerns for public health professionals worldwide and are linked to increased disability as well as mortality [1, 2]. The rise in BMI globally means that many countries are now at risk of experiencing the adverse sequelae of obesity which were previously the concern only of developed countries [3]. One of the reasons for this weight problem is low levels of physical activity, which has been linked between 3.2 million and 5.4 million deaths worldwide each year [4, 5]A lack of physical activity has also been linked to cardio-metabolic conditions such as diabetes and raised blood pressure [6, 7].

The World Health Organization (WHO) 25 by 25 agenda aims to decrease levels of physical inactivity 10 % by 2025, in order to avert large projected increases in non-communicable diseases (NCDs) [8]. In high-income countries, low levels of physical activity have been linked to growth in car ownership and consequent reductions in active travel (walking and cycling for transport). In low and middle income countries this transition appears well underway, precipitated by rising car ownership associated with increasing affluence [9, 10] as well as rapid, unplanned urbanization [11, 12]. These changes are occurring in the context of road building being prioritized as a prerequisite for economic growth with limited consideration of active travel in their planning and construction.

Raising levels of active travel is increasingly being promoted as a key action to address the growing burden of adiposity and NCDs globally [13]. A systematic review of trials and cohort studies has identified positive health effects of active travel [14], but the vast majority of included studies were conducted in high-income countries and many rely on self-reported outcome measures [14]. Although there is an increasing amount of data available on physical activity levels globally [5], a lack of research on the correlates of physical activity and active travel in low and middle-income countries has been noted [15, 16]. Such evidence can inform local and national policy makers in decisions on the relative merits of strategies to encourage active travel, and may inform efforts to combat NCDs in these settings. This study aims to (1) characterize the socio-demographic correlates of using active travel among older adults in six populous middle-income countries (2) examine whether use of active travel is associated with adiposity, systolic blood pressure (SBP) and self-reported diabetes in these countries.

## Methods

### Sample and data

This study presents secondary analysis of data from the WHO Study on Global Ageing and Adult Health (SAGE). The study collected data on six middle-income countries China, Ghana, India, Mexico, Russia and South Africa. The focus of the study was individuals over the age of 50 years although it also includes a smaller number of participants aged between 18 and 49 years [17]. The study employed a clustered household sampling strategy designed to obtain nationally representative samples from each country, with data collection between 2007 and 2010 designed to allow cross country comparisons of various indicators [18]. The study included both household and individual questionnaires administered by trained interviewers as well as nurse visits for objective measurement of key health indicators. The SAGE study has ethical approval from the WHO Ethical Review Committee and all study participants gave informed consent. Researchers can access the raw data after completing an agreement on the WHO website (http://www.who.int/healthinfo/sage/en/) and full details of the methods of SAGE are reported elsewhere [17]. The total sample size across all countries was 47,443 (China: 15,050, Ghana 5573, India: 12,198, Mexico: 4448, Russia: 4947, South Africa: 4227). 14.7 % of this total was excluded from this analysis for missing data on use of active travel or correlates, giving a final sample size of 40,477 participants.

### Variables

Adiposity outcomes for this study were Body Mass Index (BMI), overweight (BMI ≥ 25 kg/m^2^), obesity (BMI ≥ 30 kg/m^2^), high waist-to-hip ratio (≥0.90 for men or ≥0.85 for women) and waist circumference. We also examined self-reported diabetes (answers of yes to the question “*Have you ever been diagnosed with diabetes (high blood sugar)? (Not including diabetes associated with a pregnancy*)”) and systolic blood pressure (SBP), based on the mean value from three measurements.

The primary exposure of interest was constructed from the number of minutes per week participants reported walking or cycling, based on the General Physical Activity Questionnaire (GPAQ) [19] Active travel was defined based on answers to the questions “*How much time would you spend walking or bicycling for travel on a typical day?”* and *“In a typical week, on how many days do you walk or bicycle for at least 10 min continuously to get to and from places?”.* Number of days and minutes per day were combined to give a weekly value for walking and cycling, and those answering that they did not use active travel for 10 min on any day were set to zero.

For analyses of socio-demographic differences in the use of active travel we categorised participants into those using active travel for ≥150 min per week or not. This is in line with WHO guidance for both adults and older people of at least 150 min of moderate physical activity per week, in bursts of at least 10 min [20]. For analyses of associations with our health outcomes we categorised use of active travel into tertiles of exposure (minutes/week) as few participants were undertaking no active travel. Due to variation in the distributions these groups were not always equally sized. Pooled across the whole sample, these three groups were: “low/no active travel” (0–30 min/week), “moderate active travel” (31–209 min/week), and “high active travel” (210+ minutes/week).

We included data on socio-demographic characteristics: age (grouped into: 18–29, 30–39, 40–49, 50–59, 60–69, 70+ years), sex, marital status (married vs. not married), education (no formal education, less than primary education, completed primary education, completed secondary education and above), location (urban vs. rural areas), and wealth quintile. We also included data on lifestyle factors: smoking, recent alcohol use (yes/no to use in last 30 days) and fruit and vegetable consumption (≥5 portions per day vs. <5 portions per day). Minutes of moderate or vigorous work-based and leisure-based physical activity were also included.

### Analysis

Descriptive statistics were used to assess the prevalence of active travel and distributions of health outcomes in each country individually, as well as combined. Use of active travel for at least 150 min per week was examined using logistic regression. Four of the health related outcomes examined (overweight, obese, high waist-to-hip ratio and self-reported diabetes) were binary and so associations with active travel were examined using multiple logistic regression. Results are presented as Adjusted Rate Ratios (ARR) after conversion using an established method [21]. Three of the health related outcomes (waist circumference, BMI and SBP) were continuous and so multiple linear regression was used. Separate models were used for each outcome and results are presented after adjustment for the correlates of active travel mentioned above. Age was centred and treated as a continuous variable. Work-based physical activity and leisure based physical activity were combined to make a single variable for other physical activity and treated as continuous. This was partly to reduce degrees of freedom in final models and also as leisure time activity was low. Individual country analyses utilized survey weights provided by the WHO SAGE team [17] and pooled analyses utilized these as well as dummy fixed effect variables to account for differences between each country. Some individuals were missing data on certain outcomes and were not included in these analyses. Final numbers available for analysis were 39,261 (97.0 % of sample) for BMI, overweight and obesity; 39,267 (97.0 %) for waist-to-hip ratio; 38,264 (94.5 %) for waist circumference; 40,477 (100 %) for self-reported diabetes; and 39,463 (97.5 %) for systolic blood pressure. All analyses were conducted using Stata 12 software [22].

Unadjusted findings are presented in Additional file 1: Table S1.

## Results

The mean age in the pooled sample across all countries was 58.0 years old (standard deviation (SD) 14.7), and this varied from 50.0 (16.6) in India to 63.1 (14.0) in Mexico (Table 1). In the pooled sample, 38.9 % of participants had completed secondary school and above, ranging from 26.3 % in Ghana to 90.1 % in Russia. 47.4 % of the pooled sample lived in an urban area, ranging from 25.5 % in India to 75.3 % in Russia and 27.0 % of the pooled sample smoked (range 11.9 % in Ghana to 38.5 % in India).

**Table 1:** Characteristics of sample

		China	Ghana	India	Mexico	Russia	South Africa	Pooled
Age	Mean (SD)	60.3 (11.8)	60.2 (14.1)	50.0 (16.6)	63.1 (14.0)	62.4 (13.1)	60.6 (12.0)	58.0 (14.7)
Gender	Male (%)	6666 (46.5)	2623 (52.5)	4316 (38.7)	1000 (38.2)	1452 (35.5)	1306 (39.8)	17363 (42.9)
	Female (%)	7677 (53.5)	2372 (47.5)	6832 (61.3)	1618 (61.8)	2637 (64.5)	1978 (60.2)	23114 (57.1)
Marital status	Married (%)	12012 (83.7)	2986 (59.8)	8659 (77.7)	1663 (63.5)	2336 (57.1)	1668 (50.8)	29324 (72.4)
	Not married (%)	2331 (16.3)	2009 (40.2)	2489 (22.3)	955 (36.5)	1753 (42.9)	1616 (49.2)	11153 (27.6)
Education	No education (%)	3141 (21.9)	2525 (50.6)	5039 (45.2)	447 (17.1)	38 (0.9)	780 (23.8)	11970 (29.6)
	Less than primary (%)	2429 (16.9)	533 (10.7)	1162 (10.4)	967 (36.9)	71 (1.7)	793 (24.1)	5955 (14.7)
	Completed primary (%)	2811 (19.6)	622 (12.5)	1711 (15.3)	589 (22.5)	297 (7.3)	767 (23.4)	6797 (16.8)
	Completed secondary and above (%)	5962 (41.6)	1315 (26.3)	3236 (29.0)	615 (23.5)	3683 (90.1)	944 (28.7)	15755 (38.9)
Location	Rural (%)	7263 (50.6)	2942 (58.9)	8308 (74.5)	699 (26.7)	1010 (24.7)	1080 (32.9)	21302 (52.6)
	Urban (%)	7080 (49.4)	2053 (41.1)	2840 (25.5)	1919 (73.3)	3079 (75.3)	2204 (67.1)	19175 (47.4)
Wealth quartile	Q1 (lowest) (%)	2706 (18.9)	972 (19.5)	1991 (17.9)	539 (20.6)	748 (18.3)	626 (19.1)	7582 (18.7)
	Q2 (%)	2823 (19.7)	976 (19.5)	2142 (19.2)	538 (20.6)	791 (19.3)	665 (20.2)	7935 (19.6)
	Q3 (%)	2856 (19.9)	994 (19.9)	2135 (19.2)	486 (18.6)	806 (19.7)	660 (20.1)	7937 (19.6)
	Q4 (%)	2981 (20.8)	1031 (20.6)	2342 (21.0)	541 (20.7)	833 (20.4)	674 (20.5)	8402 (20.8)
	Q5 (highest) (%)	2977 (20.8)	1022 (20.5)	2538 (22.8)	514 (19.6)	911 (22.3)	659 (20.1)	8621 (21.3)
Fruit & veg consumption	Less than 5 portions per day (%)	2086 (14.5)	3481 (69.7)	9961 (89.4)	2080 (79.4)	3327 (81.4)	2415 (73.5)	23350 (57.7)
	5 or more portions per day (%)	12257 (85.5)	1514 (30.3)	1187 (10.6)	538 (20.6)	762 (18.6)	869 (26.5)	17127 (42.3)
Smoking status	Non-smoker (%)	10436 (72.8)	4400 (88.1)	6855 (61.5)	2131 (81.4)	3301 (80.7)	2406 (73.3)	29529 (73.0)
	Current smoker (%)	3907 (27.2)	595 (11.9)	4293 (38.5)	487 (18.6)	788 (19.3)	878 (26.7)	10948 (27.0)
Alcohol use	Recently used alcohol	11377 (79.3)	3425 (68.6)	10442 (93.7)	2214 (84.6)	2751 (67.3)	2781 (84.7)	32990 (81.5)
	Not recently used alcohol	2966 (20.7)	1570 (31.4)	706 (6.3)	404 (15.4)	1338 (32.7)	503 (15.3)	7487 (18.5)
Work physical activity	≤150 min per week (%)	7601 (53.0)	1339 (26.8)	3117 (28.0)	1609 (61.5)	1168 (28.6)	2066 (62.9)	16900 (41.8)
	>150 min per week (%)	6742 (47.0)	3656 (73.2)	8031 (72.0)	1009 (38.5)	2921 (71.4)	1218 (37.1)	23577 (58.2)
Leisure physical activity	≤150 min per week (%)	12996 (90.6)	4427 (88.6)	9938 (89.1)	2503 (95.6)	3766 (92.1)	3105 (94.5)	36735 (90.8)
	>150 min per week (%)	1347 (9.4)	568 (11.4)	1210 (10.9)	115 (4.4)	323 (7.9)	179 (5.5)	3742 (9.2)
Active travel level	Minimal/none	4791 (33.4)	1670 (33.4)	3800 (34.1)	1072 (41.0)	1357 (33.2)	1991 (60.6)	13,701 (33.9)
	Moderate	4106 (28.6)	1581 (31.7)	2233 (20.0)	679 (25.9)	1303 (31.9)	629 (19.2)	14,105 (34.9)
	High	5446 (38.0)	1744 (34.9)	5115 (48.9)	867 (33.1)	1429 (34.9)	664 (20.2)	12,671 (31.3)
Overall N		14343	4995	11148	2618	4089	3284	40477

Active travel level defined in three groups based on the distribution of each country or whole sample as appropriate

46.4 % of the pooled sample used active travel for at least 150 min per week (range 21.9 % in South Africa to 57.8 % in Ghana) and 58.2 % engaged in at least 150 min of work-based physical activity per week (range from 37.1 % in South Africa to 73.2 % in Ghana). Undertaking leisure-time physical activity for at least 150 min per week was not common (9.2 % pooled) and ranged from 4.4 % in Mexico to 11.4 % in Ghana.

### Socio-demographic and lifestyle characteristics and active travel

Women were less likely to use active travel for ≥150 min per week than men (ARR 0.82, 95 % Confidence Interval (CI) 0.74; 0.91 in pooled analysis) and this pattern was similar although not statistically significant in all countries (Table 2). Participants aged over 70 years were less likely to use active travel (ARR 0.71, 0.62; 0.80 vs. 18–29 year olds in pooled analysis) and this association ranged from 0.48 (0.20; 1.07) in South Africa to 0.87 (0.66; 1.07) in Ghana. Participants in the highest wealth quintile were less likely than those in the lowest quintile to use active travel for 150 min per week (ARR 0.77, 0.67; 0.88 in pooled analyses) as were those in the third and fourth highest wealth quintile.

**Table 2:** Correlates of active travel for ≥150 min per week (Adjusted Risk Ratios with 95 % Confidence Intervals)

		China	Ghana	India	Mexico	Russia	South Africa	Pooled
Age group	18–29	ref	ref	ref	ref	ref	ref	ref
	30–39	0.96 (0.71;1.24)	1.02 (0.77;1.23)	0.99 (0.89;1.08)	0.91 (0.51;1.39)	0.93 (0.60;1.19)	0.92 (0.38;1.80)	0.93 (0.81;1.06)
	40–49	0.81 (0.58;1.09)	1.01 (0.80;1.20)	1.01 (0.91;1.11)	1.09 (0.62;1.58)	0.98 (0.62;1.24)	0.89 (0.36;1.80)	0.88 (0.77;0.99)
	50–59	1.01 (0.76;1.29)	1.04 (0.85;1.22)	0.89 (0.80;0.99)	0.90 (0.42;1.49)	0.88 (0.59;1.13)	0.92 (0.42;1.69)	0.94 (0.84;1.04)
	60–69	0.93 (0.68;1.21)	0.98 (0.77;1.17)	0.82 (0.72;0.92)	0.88 (0.50;1.34)	0.67 (0.39;0.97)	0.52 (0.21;1.14)	0.86 (0.77;0.95)
	70+	0.73 (0.50;1.01)	0.87 (0.66;1.07)	0.63 (0.52;0.75)	0.65 (0.32;1.14)	0.49 (0.26;0.80)	0.48 (0.20;1.06)	0.71 (0.62;0.80)
Gender	Male	ref	ref	ref	ref	ref	ref	ref
	Female	1.00 (0.85;1.16)	0.86 (0.71;1.01)	0.55 (0.47;0.65)	0.77 (0.47;1.13)	1.00 (0.83;1.14)	0.71 (0.40;1.19)	0.82 (0.74;0.91)
Marital status	Married	ref	ref	ref	ref	ref	ref	ref
	Not married	0.98 (0.81;1.16)	1.13 (0.99;1.25)	0.89 (0.82;0.97)	1.06 (0.75;1.39)	0.95 (0.79;1.10)	1.07 (0.63;1.66)	1.04 (0.96;1.12)
Education	No education	ref	ref	ref	ref	ref	ref	ref
	Less than primary	0.95 (0.76;1.16)	0.93 (0.73;1.13)	0.92 (0.82;1.02)	1.20 (0.75;1.63)	0.89 (0.24;1.36)	1.79 (1.18;2.43)	0.92 (0.83;1.02)
	Completed primary	0.80 (0.62;1.00)	0.67 (0.50;0.86)	0.96 (0.86;1.05)	1.35 (0.91;1.73)	1.05 (0.41;1.39)	1.24 (0.65;2.03)	0.81 (0.72;0.90)
	Completed secondary and above	0.88 (0.68;1.10)	0.80 (0.63;0.97)	1.00 (0.91;1.10)	0.91 (0.50;1.40)	1.21 (0.62;1.42)	1.14 (0.58;1.91)	0.92 (0.84;1.00)
Location	Rural	ref	ref	ref	ref	ref	ref	ref
	Urban	0.49 (0.38;0.63)	1.17 (1.03;1.29)	1.14 (1.05;1.22)	1.32 (0.97;1.64)	0.98 (0.80;1.14)	1.85 (1.24;2.47)	0.94 (0.85;1.03)
Wealth quartile	1 (lowest)	ref	ref	ref	ref	ref	ref	ref
	2	0.90 (0.78;1.03)	0.89 (0.70;1.09)	0.95 (0.86;1.05)	1.25 (0.84;1.63)	1.12 (0.93;1.26)	0.82 (0.35;1.63)	0.93 (0.84;1.03)
	3	0.90 (0.75;1.05)	1.08 (0.90;1.23)	0.92 (0.82;1.01)	0.85 (0.52;1.23)	1.17 (0.98;1.30)	0.33 (0.13;0.81)	0.86 (0.77;0.95)
	4	0.77 (0.62;0.93)	0.94 (0.72;1.15)	0.90 (0.80;1.00)	0.70 (0.39;1.12)	1.11 (0.92;1.25)	0.59 (0.26;1.20)	0.84 (0.75;0.93)
	5 (highest)	0.61 (0.46;0.80)	0.80 (0.59;1.01)	0.94 (0.83;1.05)	0.96 (0.61;1.35)	1.01 (0.75;1.21)	0.59 (0.22;1.35)	0.77 (0.67;0.88)
Work physical activity	≤150 min per week	ref	ref	ref	ref	ref	ref	ref
	>150 min per week	1.40 (1.26;1.54)	1.50 (1.41;1.56)	1.31 (1.25;1.37)	1.05 (0.81;1.30)	1.32 (1.23;1.38)	2.15 (1.50;2.75)	1.38 (1.31;1.45)
Leisure physical activity	≤150 min per week	ref	ref	ref	ref	ref	ref	ref
	>150 min per week	1.32 (1.09;1.54)	1.26 (1.08;1.41)	1.20 (1.11;1.28)	1.14 (0.58;1.71)	1.13 (0.85;1.31)	1.31 (0.55;2.37)	1.35 (1.24;1.44)
Fruit & veg consumption	Less than 5 portions per day	ref	ref	ref	ref	ref	ref	ref
	5 or more portions per day	0.98 (0.76;1.22)	1.18 (1.05;1.29)	1.07 (0.97;1.16)	1.47 (1.11;1.77)	1.17 (1.04;1.27)	0.67 (0.35;1.18)	0.93 (0.85;1.01)
Smoking status	Non-smoker	ref	ref	ref	ref	ref	ref	ref
	Current smoker	0.88 (0.74;1.04)	0.94 (0.72;1.15)	1.14 (1.07;1.21)	0.99 (0.68;1.32)	0.99 (0.80;1.15)	1.03 (0.48;1.86)	1.00 (0.91;1.08)
Alcohol use in past month	Yes	ref	ref	ref	ref	ref	ref	ref
	No	1.08 (0.94;1.22)	0.89 (0.76;1.02)	0.97 (0.83;1.10)	0.78 (0.49;1.13)	1.13 (0.97;1.25)	0.78 (0.33;1.58)	1.00 (0.91;1.10)
% use AT^a^		44.9	57.8	50.6	35.7	53.3	21.9	46.4
Overall N		14343	4995	11148	2618	4089	3284	40477

^a^for ≥150 min per week

Participants who were physically active at work for ≥150 min per week were more likely to use active travel for ≥150 min per week (ARR 1.38, 1.31; 1.45 in pooled analysis) and this association was statistically significant in all countries other than Mexico (ARR 1.05, 0.81;1.30). Participants who engaged in leisure time physical activity for ≥150 min per week were also more likely to use active travel for at least 150 min per week (ARR 1.35, 1.24; 1.44 in pooled analyses). Point estimates indicated the same direction of association in all countries although findings were only statistically significant in three out of the six countries (China, Ghana and India).

### Active travel and health outcomes

Table 3 shows the mean and range for minutes of active travel in each of the active travel tertiles, as well as the means and standard deviations of health outcomes across these. Pooled across all countries the low/no active travel group had a mean of 2.0 min per week (range 0–30), the moderate group 137.3 per week (range 31–209) and the high group 463.5 per week (210+ minutes).

**Table 3:** Means (standard deviation) for selected outcomes according to categories of active travel use

Outcome (overall N)	Active travel level	China	Ghana	India	Mexico	Russia	South Africa	Pooled
Mean BMI (39,261)	Low/none	23.9 (4.0)	24.2 (6.6)	21.1 (6.5)	28.6 (6.7)	27.4 (8.9)	30.2 (9.1)	25.0 (7.3)
	Moderate	23.9 (4.1)	23.0 (5.3)	21.0 (5.8)	28.0 (5.6)	27.4 (7.5)	28.6 (7.6)	23.7 (6.0)
	High	23.9 (4.5)	22.9 (5.6)	20.7 (5.1)	27.9 (5.4)	27.6 (7.5)	28.4 (7.7)	23.6 (6.0)
% Overweight (39,261)	Low/none	33.6 (47.2)	36.8 (48.2)	16.6 (37.2)	72.7 (44.5)	67.6 (46.8)	71.8 (45.0)	42.5 (49.4)
	Moderate	33.1 (47.0)	26.5 (44.2)	14.6 (35.3)	73.9 (43.9)	69.1 (46.2)	65.4 (47.6)	33.4 (47.2)
	High	32.0 (46.6)	23.9 (42.6)	12.5 (33.1)	71.7 (45.1)	69.1 (46.2)	63.1 (48.3)	32.8 (0.5)
% Obese (39,261)	Low/none	6.0 (23.8)	14.4 (35.1)	4.7 (21.2)	36.3 (48.1)	32.7 (46.9)	43.6 (49.6)	17.3 (37.8)
	Moderate	4.7 (21.2)	8.1 (27.3)	3.4 (18.0)	31.2 (46.4)	28.9 (45.4)	36.3 (48.1)	10.2 (30.2)
	High	5.7 (23.1)	7.8 (26.9)	3.0 (17.0)	28.8 (45.3)	31.1 (46.3)	34 (47.4)	10.6 (0.3)
% High Waist to Hip Ratio (39,267)	Low/none	59.1 (49.2)	79.1 (40.6)	79.5 (40.3)	83.5 (37.1)	76.2 (42.6)	69 (46.3)	71.1 (45.3)
	Moderate	54.4 (49.8)	68.8 (46.3)	76.2 (42.6)	77.0 (42.1)	69.4 (46.1)	65.1 (47.7)	66.1 (47.3)
	High	54.5 (49.8)	74.9 (43.4)	74.8 (43.4)	81.4 (38.9)	66.5 (47.2)	58.5 (49.3)	66.7 (50.0)
Mean waist circumference (38,264)	Low/none	84.9 (10.4)	87.7 (12.9)	81 (13.1)	98.6 (13.7)	96.0 (16.6)	91.2 (22.6)	87.3 (15.5)
	Moderate	83.6 (10.2)	82.8 (12.6)	80.5 (11.6)	96.1 (12.4)	93.3 (15.8)	91.1 (21.0)	84.3 (13.0)
	High	83.8 (10.6)	82.3 (11.8)	80.2 (11.4)	95.8 (11.6)	92.7 (15.4)	87.8 (19.9)	84.2 (13.1)
Self-reported diabetes (40,477)	Low/none	6.3 (24.2)	5.0 (21.7)	6.1 (24.0)	20.1 (40.1)	10.0 (30.0)	9.8 (29.7)	8.3 (27.5)
	Moderate	5.5 (22.7)	2.9 (16.8)	4.6 (20.9)	18.7 (39.0)	6.9 (25.4)	11.1 (31.5)	6.2 (24.1)
	High	6.2 (24.0)	2.7 (16.2)	4.2 (20.0)	15.2 (35.9)	7.0 (25.5)	6.6 (24.9)	5.4 (22.6)
Mean SBP (39,463)	Low/none	143.0 (23.7)	137.6 (25.0)	121.7 (20.0)	147.6 (26.6)	144.4 (22.7)	147.2 (26.0)	139.0 (25.5)
	Moderate	140.0 (23.6)	133.1 (23.8)	120.7 (19.0)	142.5 (24.2)	140.2 (21.7)	147.5 (27.0)	133.2 (24.0)
	High	137.9 (22.7)	138.3 (25.0)	119.4 (18.2)	141.9 (23.3)	136.6 (20.1)	142.3 (23.2)	132.7 (23.2)
Mean minutes of active travel (ranges)								
Active travel level	Low/no	2.1 (0–30)	12.7 (0–80)	14.3 (0–70)	0 (0–0)	2.4 (0–49)	0.1 (0–10)	2.0 (0–30)
	Moderate	111.3 ( 31–209)	193.5 (81–350)	129.0 (71–209)	94.9 (1–175)	162.0 (50–299)	81.9 (11–179)	137.3 (31–209)
	High	396.1 (210+)	515.6 (351+)	385.4 (210+)	394.8 (176+)	50.3.4 (300+)	423.2 (180+)	463.5 (210+)

*BMI* Body Mass Index, *SBP* Systolic Blood Pressure

Pooled across all countries BMI, waist circumference and systolic blood pressure were lower and binary outcomes were all less common in the high active travel group compared to the low/no group. Across all countries mean BMIs were 25.0 kg/m^2^ for the low/no active travel group, 23.7 kg/m^2^ for the moderate group and 23.6 kg/m^2^ for the high group. These trends were evident for the individual countries, other than China (mean BMI of 23.9 kg/m^2^ across all groups) and Russia. Across all countries mean waist circumference was 87.3 cm in the low/no active travel group, 84.3 cm in the moderate group and 84.2 cm in the high group. These trends were similar across all countries as were associations for the different adiposity measures. Across all countries systolic blood pressure was lower among the high active travel group (132.7 mm/Hg) than in the moderate (133.2 mm/Hg) or the low/no group (139.0 mm/Hg).

Table 4 shows the results from fully adjusted analyses of health outcomes by tertiles of active travel use. In the pooled analysis waist circumference was lower among those in the moderate (−1.52 cm, −2.40; −0.65) and high active travel groups (−2.16 cm, −3.07; −1.26). These associations varied across the countries studied, e.g. for high active travel from −5.79 cm (−9.54; −0.48) in Russia to 0.05 cm (−0.82; 0.93) in India. BMI was lower among those in the high active travel group (−0.54 kg/m^2^, −0.98; −0.11) but not the moderate group (−0.26 kg/m^2,^ −0.62; 0.06) in pooled analyses. Associations between BMI and high use of active travel varied from −1.16 kg/m^2^ (−2.28; −0.04) in Ghana to 0.63 kg/m^2^ (−2.02; 3.28) in South Africa. Moderate (ARR 0.79, 0.68; 0.92) and high (ARR 0.71, 0.61; 0.84) use of active travel was associated with reduced waist-to-hip ratio in pooled analyses. These associations varied across countries e.g. for high active travel from ARR 0.33 (0.17; 0.67) in South Africa to ARR 1.18 (0.62; 2.24) in Mexico. Both moderate (−1.63 mm/Hg (−3.19; −0.06) and high (−2.33 mm/Hg (−3.98; −0.69) use of active travel was associated with lower systolic blood pressure. This pattern was inconsistent across countries studied.

**Table 4:** Adjusted associations between active travel and selected health outcomes

	Active Travel Level^a^	Overweight (BMI ≥ 25)	Obese (BMI ≥ 30)	High WHR	Waist circumference	BMI	Self-reported Diabetes	SBP
China	Moderate	1.07 (0.83 ; 1.37)	0.95 (0.56 ; 1.59)	0.92 (0.73 ; 1.15)	0.27 (−0.94 ; 1.48)	−0.15 (−0.64 ; 0.34)	1.31 (0.84 ; 2.06)	−4.15 (−6.96 ;–1.34)
	High	0.91 (0.71 ; 1.16)	1.12 (0.64 ; 1.97)	0.76 (0.61 ; 0.94)	−0.84 (−1.92 ; 0.22)	−0.19 (−0.63 ; 0.26)	1.26 (0.79 ; 1.00)	−4.40 (−7.33 ;–1.46)
Ghana	Moderate	0.68 (0.47 ; 0.99)	0.79 (0.47 ; 1.34)	0.65 (0.43 ; 0.97)	−3.24 (−5.22 ;–1.47)	−0.96 (−1.79 ;–0.13)	1.31 (0.51 ; 3.35)	−1.30 (−4.03 ; 1.42)
	High	0.40 (0.27 ; 0.61)	0.75 (0.40 ; 1.42)	1.11 (0.71 ; 1.73)	−3.28 (−5.22 ;–1.33)	−1.16 (−2.28 ;–0.04)	1.81 (0.78 ; 7.17)	3.37 (0.07 ; 6.67)
India	Moderate	1.07 (0.81 ; 1.42)	0.88 (0.58 ; 1.33)	1.06 (0.84 ; 1.35)	−0.14 (−1.08 ; 0.80)	0.17 (−0.40 ; 0.75)	0.65 (0.38 ; 1.10)	0.27 (−1.23 ; 1.80)
	High	0.78 (0.61 ; 0.99)	0.82 (0.57 ; 1.17)	1.09 (0.89 ; 1.34)	0.05 (−0.82 ; 0.93)	−0.13 (−0.56 ; 0.30)	0.66 (0.37 ; 1.19)	−0.56 (−2.01 ; 0.89)
Mexico	Moderate	0.58 (0.29 ; 1.18)	0.67 (0.33 ; 1.35)	0.70 (0.32 ; 1.49)	−2.50 (−6.27 ; 1.28)	−1.50 (−3.04 ; 0.04)	0.95 (0.37 ; 2.42)	−2.49 (−7.06 ; 2.08)
	High	1.17 (0.65 ; 2.09)	0.88 (0.42 ; 1.85)	1.18 (0.62 ; 2.24)	−2.25 (−5.92 ; 1.41)	−0.63 (−2.11 ; 0.85)	1.19 (0.57 ; 2.49)	−0.68 (−5.27 ; 3.92)
Russia	Moderate	1.34 (0.85 ; 2.13)	1.15 (0.61 ; 2.17)	0.73 (0.42 ; 1.29)	−5.21 (−9.95 ;–0.48)	0.86 (−0.71 ; 2.42)	0.72 (0.33 ; 1.59)	3.38 (−0.03 ; 6.79)
	High	0.70 (0.41 ; 1.19)	0.93 (0.47 ; 1.49)	0.58 (0.36 ; 0.94)	−5.79 (−9.54 ;–2.04)	0.09 (−1.62 ; 1.81)	0.71 (0.29 ; 1.74)	−2.82 (−5.95 ; 0.32)
South Africa	Moderate	0.46 (0.25 ; 0.87)	0.63 (0.36 ; 1.13)	0.52 (0.26 ; 1.04)	−5.49 (−10.98 ; 0.00)	−1.85 (−3.76 ; 0.06)	1.32 (0.69 ; 2.54)	6.81 (0.57 ; 13.05)
	High	0.94 (0.45 ; 1.98)	1.09 (0.53 ; 1.13)	0.33 (0.17 ; 0.67)	−1.66 (−7.29 ; 3.98)	0.63 (−2.02 ; 3.28)	1.01 (0.50 ; 2.00)	3.59 (−1.90 ; 9.07)
Pooled	Moderate	0.88 (0.75 ; 1.03)	0.91 (0.70 ; 1.18)	0.79 (0.68 ; 0.92)	−1.52 (−2.40 ;–0.65)	−0.26 (−0.62 ; 0.09)	1.14 (0.87 ; 1.51)	−1.63 (−3.19 ;–0.06)
	High	0.71 (0.59 ; 0.86)	0.82 (0.62 ; 1.08)	0.71 (0.61 ; 0.84)	−2.16 (−3.07 ;–1.26)	−0.54 (−0.98 ;–0.11)	0.99 (0.73 ; 1.33)	−2.33 (−3.98 ;–0.69)

^a^Reference group = low/no active travel group

Figures in bold are statistically significant at *p* ≤ 0.05. *BMI* Body Mass Index, *SBP* Systolic Blood Pressure, *WHR* Waist-to-Hip-Ratio

Results adjusted for mean centred age, sex, marital status, education, location, household wealth quintile, minutes of vigorous and moderate physical activity, fruit and vegetable consumption, smoking status and alcohol use

**Fig 1:**
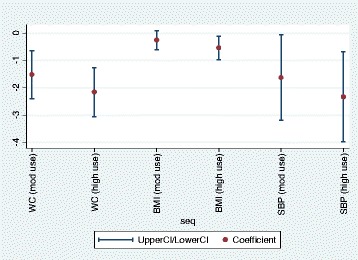
Active travel and selected health outcomes

Figure 1 presents findings of these analyses for the continuous health outcomes graphically. This shows that although point estimates were lower for high use of active travel than moderate use, overlapping confidence intervals do not conclusively suggest a dose-response relationship.

Additional file 1: Table S1 shows the results of our unadjusted analyses. Compared with the fully adjusted analyses, larger associations between active travel and health outcomes were identified suggesting some attenuation when controlling for socio-demographic and lifestyle factors. E.g. in pooled analyses, moderate use of active travel was associated with a larger reduction in waist circumference (−2.17 cm, −3.11; −1.24) than in fully adjusted analyses (−1.52 cm, −2.40; −0.65).

## Discussion

With nationally representative data for older adults from six middle-income countries this study has found that there was wide variation in use of active travel for ≥150 min per week, from 21 % in South Africa to 58 % in Ghana. Older people were less likely to use active travel for 150 min per week, as were women and those with higher levels of household wealth. High use of active travel was associated with reduced risks of being overweight, having a high waist-to-hip ratio, as well as a lower waist circumference, BMI and systolic blood pressure. Moderate use of active travel was associated with reduced risks of having a high waist-to-hip ratio and lower waist circumference and systolic blood pressure. The findings were broadly consistent across the countries studied, although this was not universal. These findings are based on objective measurements of these health outcomes, which has been noted as a limitation in previous studies of active travel [23, 24].

The findings identified here are consistent with data from high-income settings as well as a growing evidence base on the impacts of active travel in low and middle income settings [25, 26]. For example, a recent systematic review has highlighted the positive impacts of active travel [14] and a previous review concluded that use of active travel to travel work was associated with an 11 % reduction in cardiovascular risk [27]. The few studies in middle-income countries have generally suggested a positive impact of use of active travel [26, 28].

This study has a number of strengths and limitations. The sampling strategy of SAGE designed to produce nationally representative estimates. All of the outcomes apart from diabetes used were based on nurse measurements rather than self-reports. There is ongoing debate over the validity of different measures of adiposity, particularly for use in cross-national comparisons [29]. Our study sought to address this concern by using three separate measures of adiposity (BMI, waist-to-hip ratio and waist circumference). For these reasons, we opted not to utilize country-specific cut-points to define overweight and obesity. The various measures used here gave broadly similar findings which strengthens the case for the health benefits of active travel. This study examines walking and cycling for any purpose, and so should capture trips for any reason, as opposed to focusing only on the journey to work or school which is common in some previous work [14].

Data on use of active travel however, was based on self-report and may be subject to recall bias. As with all cross-sectional studies there remains the possibility that some of the associations found here were due to reverse causality [30], and although we controlled for a wide range of possible confounders, residual confounding remains a possibility. Factors such as exposure to air pollution and particulate matter may have had an impact on the results for blood pressure and diabetes [31] although as this is likely to be socially patterned and should be partly controlled for by adjustment for wealth and education. This study used tertiles of exposure (minutes/week) as few participants were undertaking no active travel. Therefore those in the reference group were undertaking some active travel (up to 30 min per week in pooled analyses) which may have led to an underestimation of any associations between active travel and health outcomes. Additionally, this study was unable to examine factors likely to play a role in transport decisions, such as availability of cars, roads, pavements or public transport. These factors are likely to vary across the countries included. Longitudinal studies are needed to confirm these findings and investigate causal processes. We were also unable to examine the impact of the use of active travel on road traffic accidents, which remain a significant cause of mortality and morbidity globally, particularly in India [32].

Rising levels of obesity and the associated cardio-metabolic problems present a large threat to the health and development of middle-income countries [33]. The associations between active travel and health outcomes identified here could be important to population health and their potential for benefits compares well with other strategies for managing adiposity and NCDs. For example, the 0.5 kg/m^2^ reduction in BMI associated with high use of active travel compares favorably with many individually focused interventions such as dietary interventions or web-based interventions to increase physical activity [34]. Additionally, the reduction of 2.3 mmHg in systolic blood pressure among those in the high active travel group suggests that use of active travel may be important in shifting the distribution of one of the largest risk factors for mortality globally [4]. For example, a 2 mm/Hg reduction in population systolic blood pressure has been estimated to reduce strokes by 10 % and Ischemic Heart Disease by 7 % [35]. Although this study did not test an intervention these findings, combined with those from previous studies, suggest that increasing active travel should be integral to strategies aiming to reduce the burden of obesity and non-communicable disease in middle-income countries. Our study also finds that leisure time physical activity is low in these settings, a finding which has been echoed in previous work [36]. Our findings strengthen the WHO call to develop policy measures which promote the incorporation of physical activity into daily life, such as active travel [37]. Measures to encourage active travel include the re-engineering of urban areas to facilitate walking and cycling as well as increases in gasoline taxes and subsidized bicycle purchase schemes [38].

Future research is needed to examine the effectiveness of interventions to increase active travel in low and middle-income country settings. Full evaluation of the costs and benefits of active travel will need to consider not only the health outcomes examined here, but also the possibility of deleterious effects of exposure to air pollution and road traffic accidents. Strategies to increase active travel at the expense of motorized transport can additionally have positive longer term impacts on carbon emissions [39] as well as the more immediate health benefits identified here. These potential benefits need to better quantified in order to inform policy development in this area.

## Additional file

Additional file 1: Table S1. Unadjusted associations between active travel and health outcomes.
