# Supplementary material for: Associations between active travel and weight, blood pressure and diabetes in six middle income countries: a cross-sectional study in older adults
Source: Int J Behav Nutr Phys Act. 2015 May 20;12:65. doi: 10.1186/s12966-015-0223-3 (PMC4443597; doi:10.1186/s12966-015-0223-3)
Supplement: Additional file 1: Table S1. — Unadjusted associations between active travel and health outcomes. [file 12966_2015_223_MOESM1_ESM.doc]

Additional file 1 Table S1 Unadjusted associations between active travel and health outcomes

|  | Active Travel Level* | Overweight (BMI ≥ 25) | Obese (BMI ≥ 30) | High WHR | Waist circumference | BMI | Self-reported Diabetes | SBP |
| --- | --- | --- | --- | --- | --- | --- | --- | --- |
| China | Moderate | 1.03 (0.87 ; 1.21) | 0.98 (0.56 ; 1.66) | 0.96 (0.88 ; 1.05) | **−0.22 (−1.54 ; 1.10)** | −0.20 (−0.76 ; 0.36) | 1.12 (0.73 ; 1.71) | **−5.07 (−8.19 ;−1.95)** |
|  | High | 0.92 (0.77 ; 1.08) | 1.14 (0.66 ; 1.95) | 0.90 (0.88 ; 1.05) | **−1.33 (−2.46 ;−0.20)** | −0.26 (−0.77 ; 0.25) | 1.28 (0.82 ; 1.97) | **−5.98 (−9.21 ;−2.76)** |
| Ghana | Moderate | **0.66 (0.50 ; 0.85)** | **0.60 (0.39 ; 0.92)** | **0.84 (0.73 ; 0.94)** | **−5.27 (−7.33 ;−3.20)** | **−1.80 (−2.73 ;−0.87)** | 0.84 (0.43 ; 1.31) | **−2.94 (−5.79 ;−0.09)** |
|  | High | **0.44 (0.32 ; 0.59)** | **0.50 (0.29 ; 0.84)** | 0.95 (0.86 ; 1.03) | **−6.42 (−8.48 ;−4.36)** | **−2.30 (−3.41 ;−1.18)** | 0.80 (0.41 ; 1.27) | 1.43 (−1.92 ; 4.78) |
| India | Moderate | 0.94 (0.74 ; 1.18) | 0.77 (0.51 ; 1.15) | 0.97 (0.92 ; 1.02) | −0.54 (1.55 ; 0.46) | −0.05 (−0.65 ; 0.55) | **0.63 (0.40 ; 0.97)** | −0.14 (−1.79 ; 1.51) |
|  | High | **0.63 (0.51 ; 0.78)** | **0.59 (0.41 ; 0.83)** | **0.93 (0.88 ; 0.97)** | −0.91 (−1.83 ; 0.01) | **−0.59 (−1.04 ;−0.14)** | **0.59 (0.39 ; 0.90)** | **−1.57 (−2.95 ;−0.18)** |
| Mexico | Moderate | 0.88 (0.61 ; 1.10) | 0.87 (0.35 ; 1.65) | 0.96 (0.76 ; 1.08) | **−1.55 (−6.08 ; 2.97)** | −1.40 (−3.50 ; 0.37) | 0.85 (0.36 ; 1.73) | −2.78 (−9.38 ; 3.81) |
|  | High | 1.10 (0.94 ; 1.21) | 0.90 (0.53 ; 1.36) | 1.07 (0.97 ; 1.13) | **−1.21 (−4.78 ; 2.37)** | −0.70 (−2.28 ; 0.88) | 1.06 (0.53 ; 1.89) | −0.03 (−5.54 ; 5.47) |
| Russia | Moderate | 0.90 (0.71 ; 1.06) | 0.80 (0.48 ; 1.23) | **0.73 (0.56 ; 0.89)** | **−9.81 (−14.89 ;−4.74)** | −0.97 (−3.24 ; 1.30) | **0.43 (0.22 ; 0.84)** | **−7.08 (−12.42 ;−1.74)** |
|  | High | 0.75 (0.47 ; 1.02) | **0.60 (0.36 ; 0.94)** | **0.69 (0.44 ; 0.92)** | **−10.56 (−16.12 ;−5.00)** | −1.21 (−3.18 ; 0.77) | **0.41 (0.23 ; 0.74)** | **−12.33 (−19.64 ;−5.01)** |
| South Africa | Moderate | **0.75 (0.50 ; 0.98)** | **0.65 (0.42 ; 0.95)** | 0.76 (0.50 ; 1.01) | **−8.61 (−14.87 ;−2.34)** | **−3.03 (−5.00 ;−1.05)** | 0.84 (0.45 ; 1.53) | 6.80 (−0.75 ; 14.36) |
|  | High | 0.93 (0.65 ; 1.14) | 0.88 (0.54 ; 1.29) | **0.54 (0.32 ; 0.81)** | −3.40 (−8.92 ; 2.12) | −0.74 (−3.41 ; 1.93) | 0.59 (0.30 ; 1.14) | 4.38 (−2.48 ; 11.23) |
| Pooled | Moderate | **0.89 (0.80 ; 0.98)** | 0.83 (0.65 ; 1.04) | **0.91 (0.86 ; 0.96)** | **−2.17 (−3.11 ;−1.24)** | **−0.45 (−0.84 ;−0.07)** | 0.91 (0.70 ; 1.17) | **−2.75 (−4.41 ;−1.09)** |
|  | High | **0.76 (0.67 ; 0.86)** | **0.68 (0.53 ; 0.87)** | **0.85 (0.79 ; 0.90)** | **−2.99 (−3.97 ;−2.00)** | **−0.95 (−1.42 ;−0.49)** | **0.72 (0.55 ; 0.95)** | **−3.39 (−5.53 ;−1.66)** |

* Reference group = low/none active travel group

Figures in bold are statistically significant at p ≤ 0.05. BMI = Body Mass Index, SBP = Systolic Blood Pressure, WHR = Waist-to-Hip-Ratio

Adjusted results adjusted for mean centred age, sex, marital status, education, location, household wealth quintile, minutes of vigorous and moderate physical activity, fruit and vegetable consumption, smoking status and alcohol use
